# Supplementary material for: Transposable element-initiated enhancer-like elements generate the subgenome-biased spike specificity of polyploid wheat
Source: Nat Commun. 2023 Nov 17;14:7465. doi: 10.1038/s41467-023-42771-9 (PMC10656477; doi:10.1038/s41467-023-42771-9)
Supplement: Supplementary file 1 — Supplementary Information [file 41467_2023_42771_MOESM1_ESM.pdf]

Transposable element-initiated enhancer-like elements generate the subgenome-biased spike specificity of polyploid wheat

Xie *et al.*

Supplementary Note 1. Profiling of genome-wide TSS associated with common wheat development

The CAGE-seq signals were highly enriched in the annotated TSS (Supplementary Fig.2a). The genomic tracks (Supplementary Fig.2b–c) revealed the TSS clusters coincide well with the annotated TSSs and the newly identified alternative TSSs of coding genes based on RNA-seq. On the basis of their local distribution, TSSs were classified as broad or sharp types (Supplementary Fig.2d–e), with sharp TSSs dependent on the TATA box (Supplementary Fig.2f–g). The sharp TSS-initiated genes were generally associated with environmental responses and plant cell wall organization, whereas the broad TSS-initiated genes were highly expressed and were related to housekeeping functions, including photorespiration and proteasome assembly (Supplementary Fig.2h–i and Supplementary Fig.3). These results are consistent with previous reports indicating that distinct TSS profiles differentially affect plant development[1-4].

Alternative TSSs often influence the development of mammals[5] and plants[6-8]. The CAGE-seq analysis in this study detected 7,340 genes with multiple TSSs (Supplementary Fig.4a), most of which were not annotated in reference gene models. Three of four randomly selected TSSs were validated by 5′ RACE (Supplementary Fig.4b-d and Supplementary Fig.5). For genes with multiple TSSs, the first TSS generally produced the most abundant transcripts (Supplementary Fig.4c). Functional analyses demonstrated that multiple TSS-driven genes were mostly involved in environmental responses (Supplementary Fig.6). This is consistent with the results of a recent study on wheat, which indicated that defense-related genes are regulated by a complex mechanism involving multiple distal regulatory elements (REs)[9]. We observed that alternative TSSs resulted in diverse products, the addition of a new signal peptide (Supplementary Fig.4b), and altered uORF transcription (Supplementary Fig.4d), which may affect translational efficiency as well as protein functions and subcellular localization. Some of the alternative TSSs were heterogeneous across subgenomes (Supplementary Fig.7). The comparison of homeologous genes suggested that differential presence of uORFs is associated with the variability in the mRNA abundance across subgenomes (Supplementary Fig.8). Thus, the comprehensive genome-scale TSS atlas and epigenetic signatures are useful resources for studies on regulatory specificity across subgenomes in common wheat.

Supplementary Note 2. Genetic and epigenetic features distinguishing ELE and gene TSSs

Unlike TSS-initiated wheat genes enriched with H3K36me3 and H3K4me1, the ELE transcripts had relatively few H3K36me3 and H3K4me[9,10] (Supplementary Fig.11a). Besides epigenetic features, we examined genetic similarities and differences between gene and ELE TSSs. In terms of the transcriptional direction, unlike the balanced bidirectional transcription of mammalian enhancers[11,12], ELEs and genes in common wheat were strongly biased for the sense direction (Supplementary Fig.11b-c). Considering the TSS sequence largely determines transcription factor (TF) binding specificity, we searched for differentially enriched TF-binding motifs in TSSs among ELEs and genes (Supplementary Fig.12). The AG- and AT-rich motifs, including BPC, WRKY, GATA, and MYB TF-binding motifs, were more frequently present around ELE TSSs, whereas CG-rich motifs, including PI-, Dof-, and AP1-binding sites, were more common surrounding coding gene TSSs (Supplementary Fig.12). We used a support vector machine (SVM) framework[13] to investigate whether these two TSS types can be distinguished according to sequence k-mers alone (see Methods). An AUROC of 86.85 was obtained, indicating the TSS sequence diversity can explain most of the difference in the initiation of transcription between coding genes and ELEs in common wheat (Supplementary Fig.13a), suggestive of the regulatory significance of TSS sequences.

We next used the SVM framework to examine the TSSs indistinguishable between ELEs and genes, which revealed the considerable similarity between the ELE and gene TSSs. To investigate the functional implications of these ELEs, we linked ELEs and genes (distance <2 Mb) according to the correlated CAGE signals and identified ELE–gene pairs with homologous TSS sequences (Supplementary Fig.13b-c and Supplementary Data 3). Using Hi-C data to examine the physical interactions, we detected an apparent positive association between chromatin contact frequency and the sequence similarity of ELE–gene pairs (Supplementary Fig.13d). Hence, we elucidated the genetic and epigenetic features distinguishing ELE and gene TSSs, while also determining that a small proportion of ELEs and target genes have highly similar TSS sequences, possibly indicating the high likelihood of looping and concerted regulation.

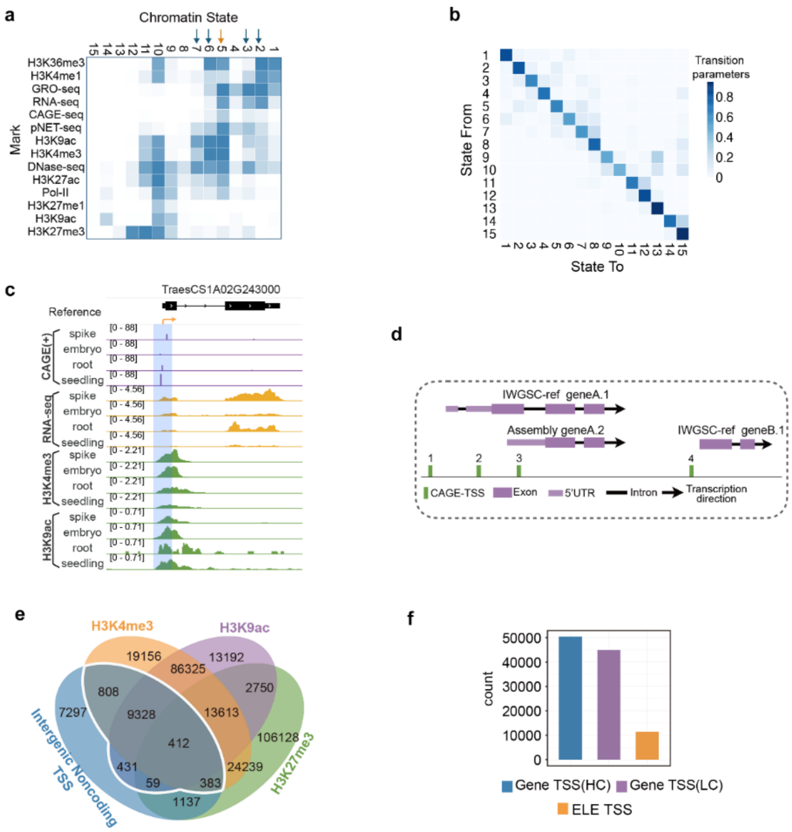

Supplementary Figure 1. Workflow for TSS-like chromatin state generation and CAGE-TSS annotation.

a. Chromatin states determined by a multivariate hidden Markov model and CAGE-seq, nascent RNA-seq, mRNA-seq, and ChIP-seq (epigenetic markers) data for seedlings. The heatmap presents the emission parameters; columns and rows correspond to different states and markers, respectively. The identified TSS-like states are presented in (d). The red and blue arrows indicate TSS-like states and transcription elongation states, respectively. CAGE-signals in state 5 with a relatively low read density surrounding gene TSSs and a lack of CAGE-clusters were used as the criteria for annotating low-confidence gene TSSs.
b. The transition parameters for each state. Colors indicate state transition possibilities from the y-axis to the x-axis.
c. Genomic tracks illustrate the CAGE signal and epigenetic pattern of a gene with TSS annotated by chrHMM.
d. Illustration of the assigned CAGE-TSSs. The CAGE-TSSs located near the 5′ UTR of ref-genes (geneA.1 and geneB.1) or a *de novo* assembled gene (geneA.2) were defined as TSSs for the given gene. CAGE-TSS 1–4 belonged to transcripts A.1, A.1, A.2, and B.1, respectively.
e. Venn diagram showing the overlap between intergenic TSSs and regulatory elements (REs) reflected by the enrichment of H3K9ac, H3K4me3, or H3K37me3. Intergenic CAGE-TSSs located in a region enriched with H3K9ac or H3K4me3 (white circle) were defined as ELE TSSs.
f. Number of gene and ELE TSSs detected and annotated by CAGE-seq. HC, high-confidence; LC, low-confidence.

Source data are provided as a Source Data file.

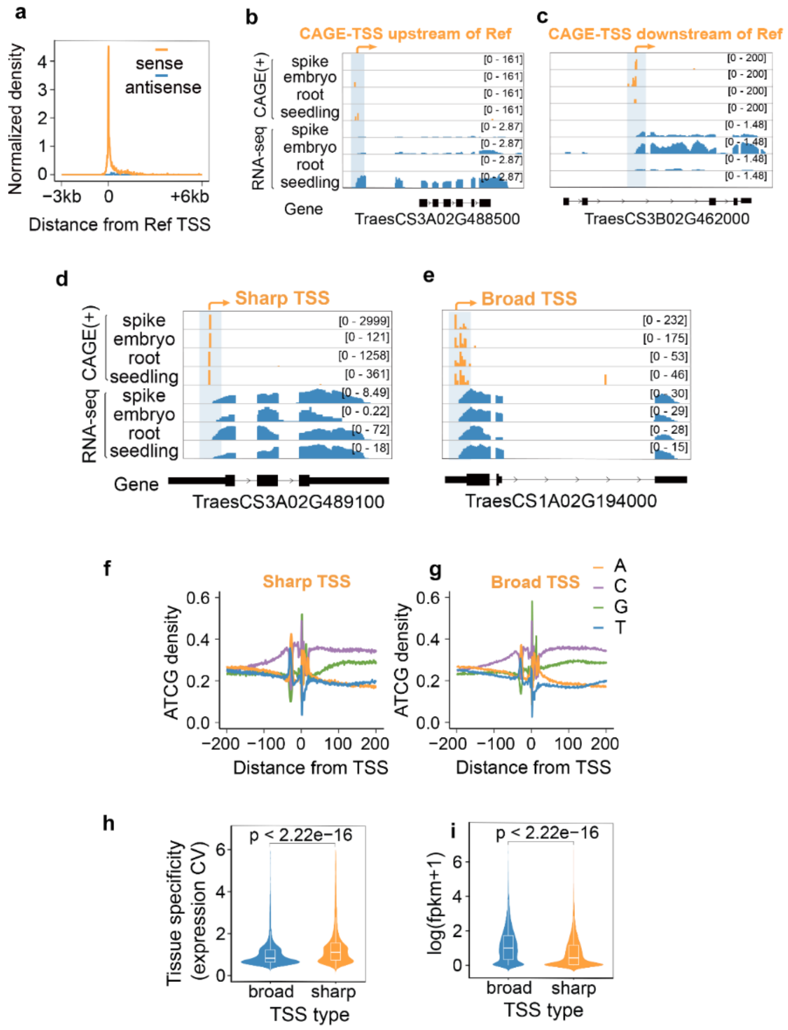

Supplementary Figure 2. The CAGE-seq analysis detected different TSS types in the coding genes in common wheat.

a. Average CAGE-seq read density around annotated TSSs. Sense and antisense read densities were plotted separately.

b–c. Genomic tracks illustrating CAGE-TSSs upstream (b) and downstream (c) of the annotated TSS (IWGSC Ref1.1). The results of the 5′ RACE validation are presented in Supplementary Fig.5.

d–e. Genomic tracks illustrating the CAGE signals and transcription from sharp and broad TSSs.

f–g. Average nucleotide frequencies of the sharp (f) and broad (g) TSSs.

h. Tissue specificity of the genes with sharp (n =9499) or broad (n = 25705) promoters as determined by expression variations (CV) across tissues. Significance was determined according to the two-tailes Welch two-sample t-test. Horizontal lines in boxplots show median, hinges show IQR, whiskers show 1.5 × IQR, points beyond 1.5 × IQR past hinge are shown.

i. Expression levels of genes with sharp (n =9499) or broad (n = 25705) promoters. Significance was determined according to the two-tailes Welch two-sample t-test. Horizontal lines in boxplots show median, hinges show IQR, whiskers show 1.5 × IQR, points beyond 1.5 × IQR past hinge are shown.

Source data are provided as a Source Data file.

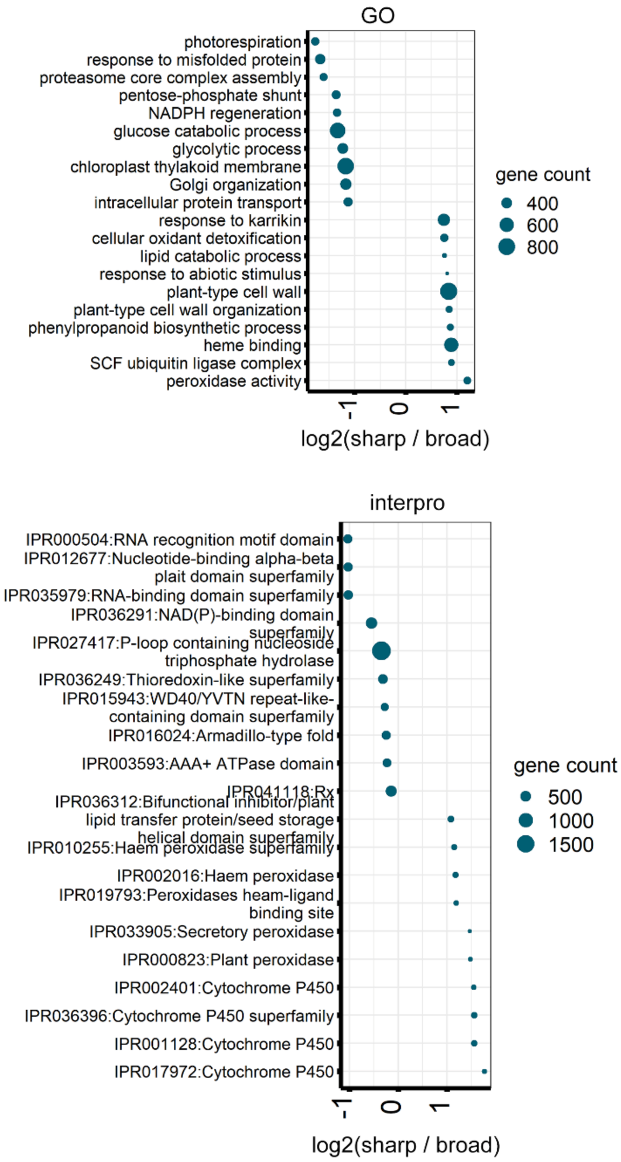

Supplementary Figure 3. GO functional terms and Interpro protein domains preferentially enriched for genes with sharp or broad TSS, respectively. The relative enrichment between genes with sharp and broad TSS were calculated. Source data are provided as a Source Data file.

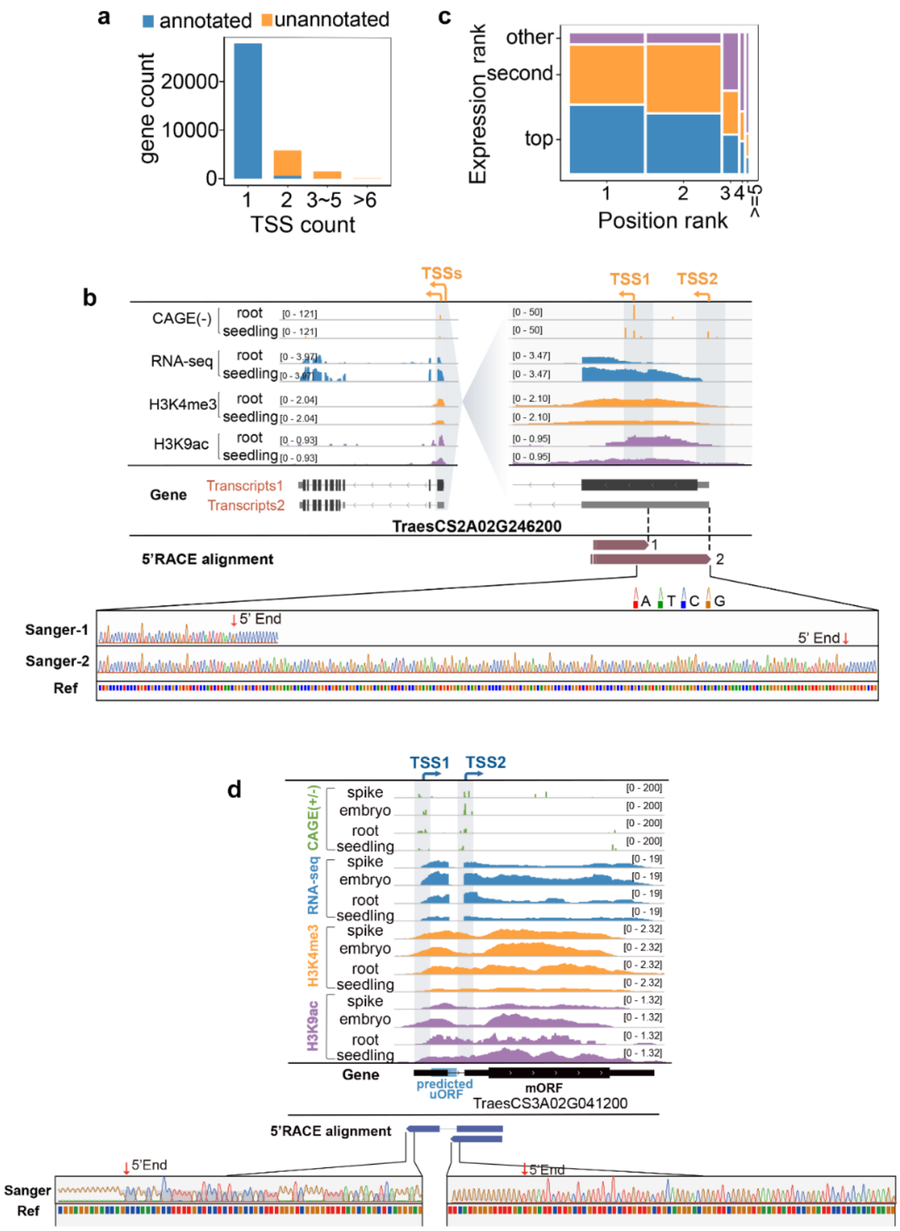

Supplementary Figure 4. The CAGE-seq detected alternative TSSs in the coding genes

a. Number of genes with single or multiple TSSs.

b. Genomic tracks illustrating the different ORFs driven by alternative TSSs at one gene locus, which were predicted by integrating CAGE and epigenetic profiles and validated by 5′ RACE.

c. Average expression level of genes driven by alternative TSSs. The x-axis presents the order of TSSs for the isoforms of the same gene from 5’ to 3’. The y-axis presents the ranked expression level for each isoform.

d. Genomic tracks illustrate the upstream ORF (uORF) predicted by integrating CAGE signals and epigenetic profiles. mORF represents the main ORF. The results of the 5′ RACE validation are presented below.

Source data are provided as a Source Data file.

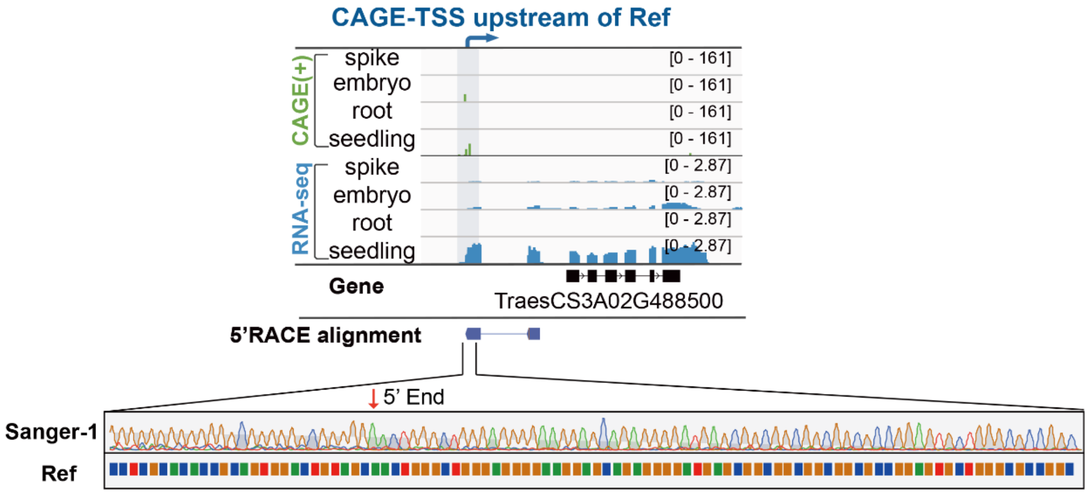

Supplementary Figure 5. Genomic tracks illustrate 5'RACE validation of a CAGE-TSS upstream of the annotated genes.

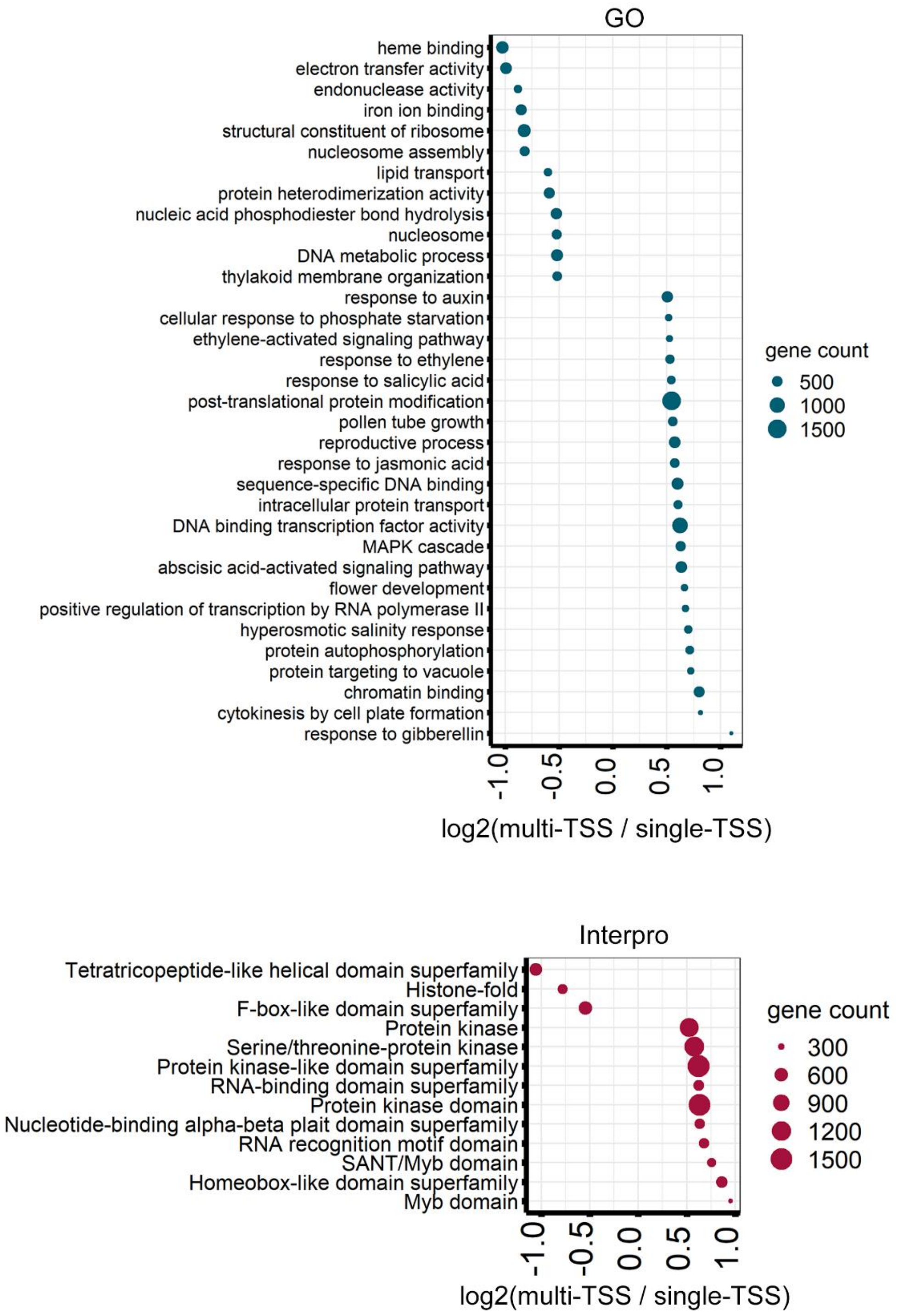

Supplementary Figure 6. Relative enrichment of GO functional terms and Interpro protein domains for genes with multiple- or single-TSS. Source data are provided as a Source Data file.

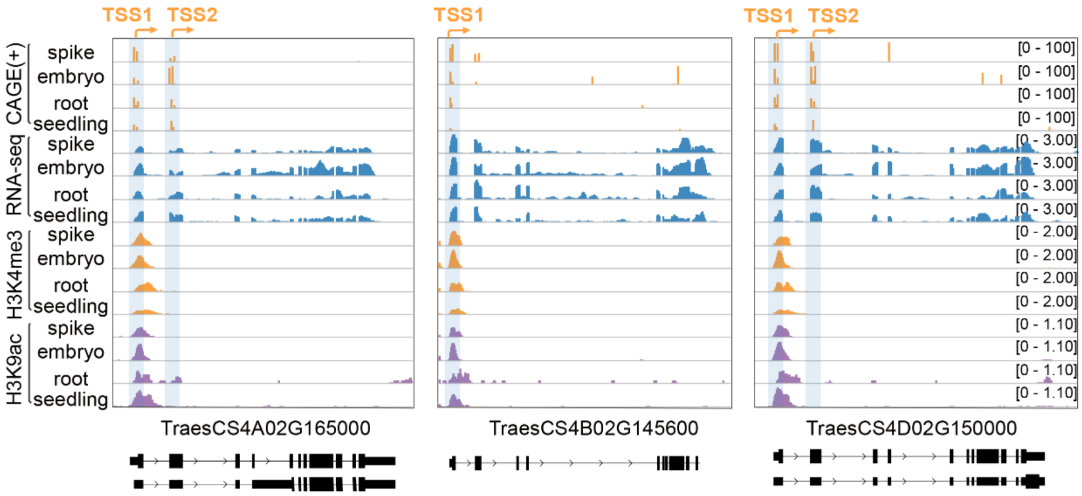

Supplementary Figure 7. Genomic tracks illustrate the CAGE signals and epigenetic signitures of a triad group with heterogeneous alternative TSSs annotated by CAGE.

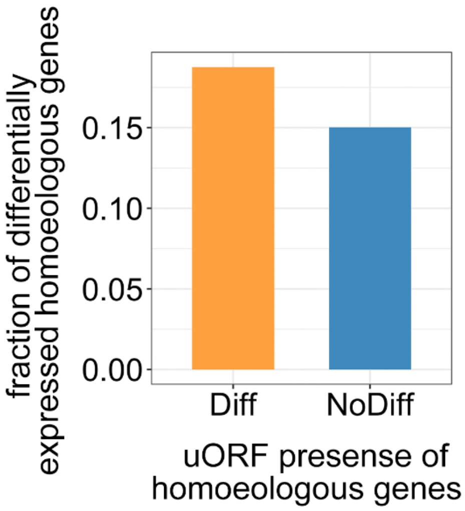

Supplementary Figure 8. Bar plot showing the ratio of differentially expressed pairs of homoeologous genes with the same or different presence of uORFs. "Diff" means that one homoeologous gene has uORF and the other does not. "NoDiff" means that homoeologous gene pairs both have uORFs or neither contains uORF. Source data are provided as a Source Data file.

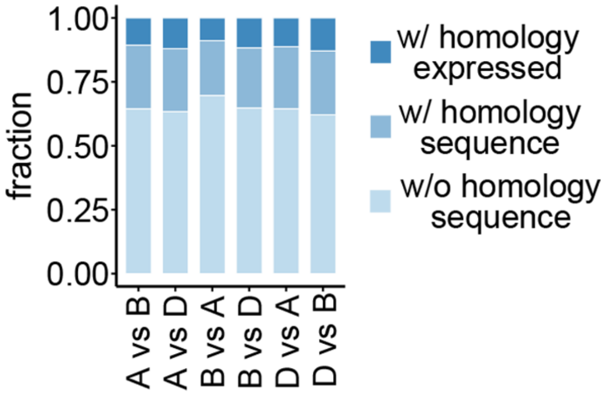

Supplementary Figure 9. Pairwise comparison of intergenic TSS clusters in col-linear regions between subgenomes. The analyses resulted in the following three cases: conserved RNA transcribed in the other subgenome, conserved DNA sequence that was not transcribed, and no sequence conservation of given TSS. Source data are provided as a Source Data file.

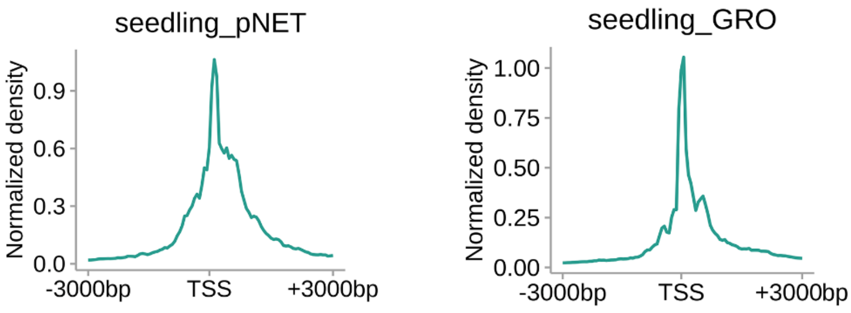

Supplementary Figure 10. Distribution of pNET-seq and GRO-seq signal intensity 3 kb upstream and downstream of ELE-TSS. Source data are provided as a Source Data file.

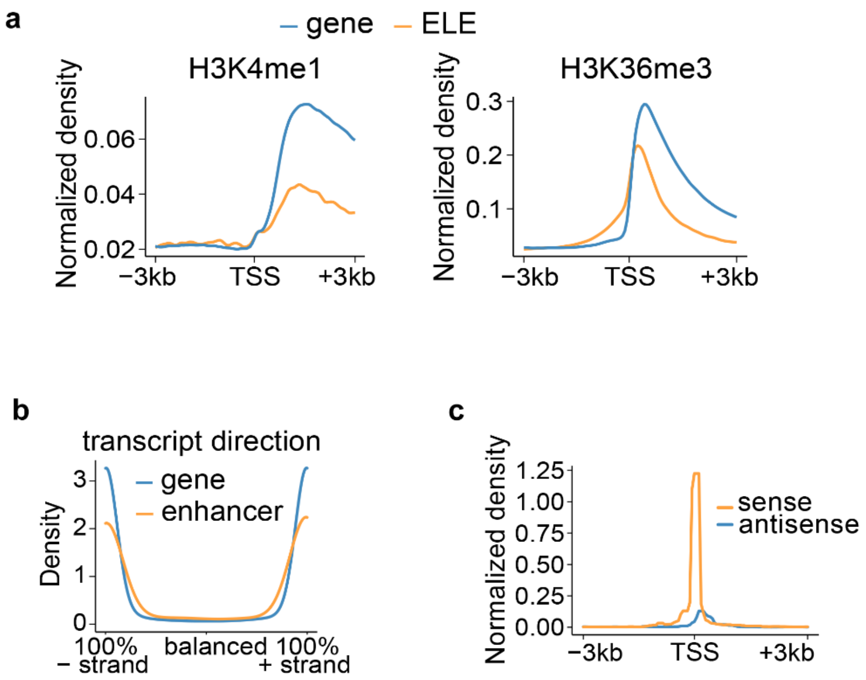

Supplementary Figure 11. The epigenetic and transcription direction of gene- and ELE-TSSs.

a. Distribution of histone modifications 3 k b up- and down-stream of gene-TSSs and ELE-TSSs.
b. Density plot illustrating the transcription direction of genes and ELEs.
c. Average CAGE-seq read density around ELE-TSSs. The CAGE signals for the antisense and sense sequences are presented separately.

Source data are provided as a Source Data file.

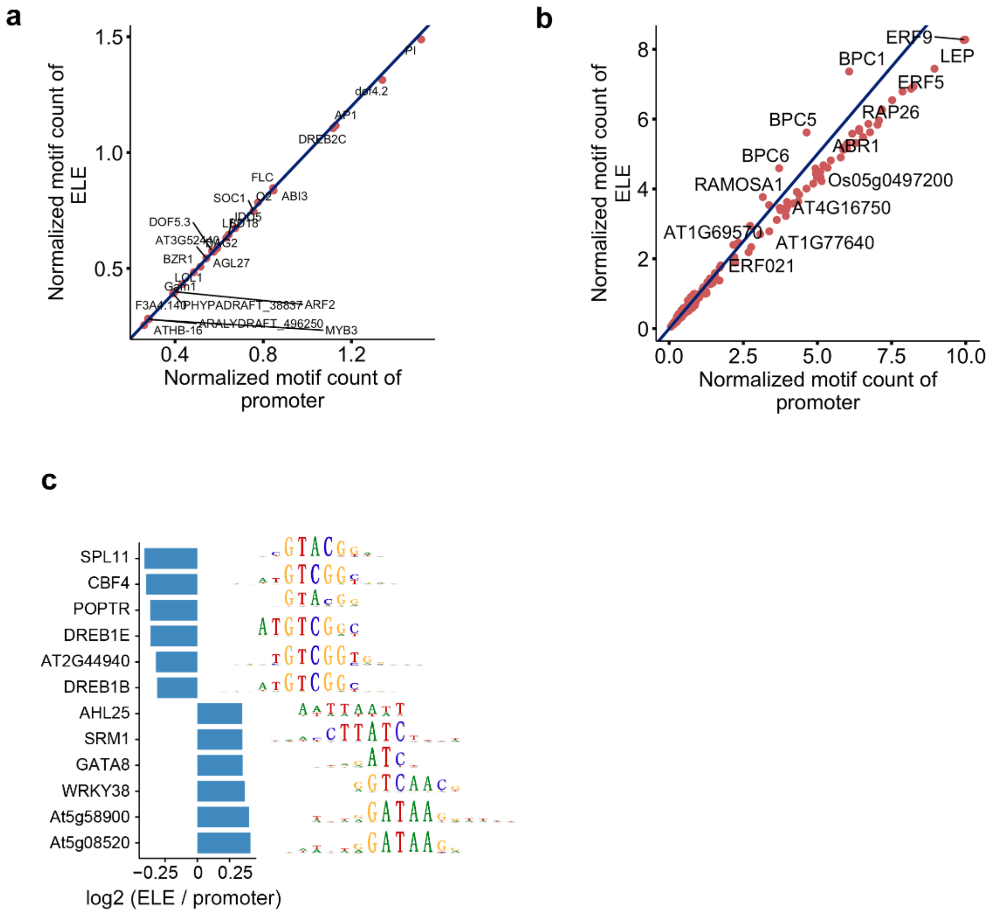

Supplementary Figure 12. Conserved and differential motif enrichment around gene- and ELE-TSSs.

a. Dot plot showing the abundance of motifs similarly enriched between gene- and ELE-TSS (1 kb up- and down-stream of TSS). Motifs with log2-transition ratios of normalized counts between ELEs and genes less than 0.03 are shown.
b. Dot plot showing the abundance of motifs differentially enriched between gene- and ELE-TSS (1 kb up- and down-stream of TSS). Motifs with higher differential abundance between ELEs and promoters are labeled.
c. Differential enrichment of motifs surrounding gene and ELE TSSs.

Source data are provided as a Source Data file.

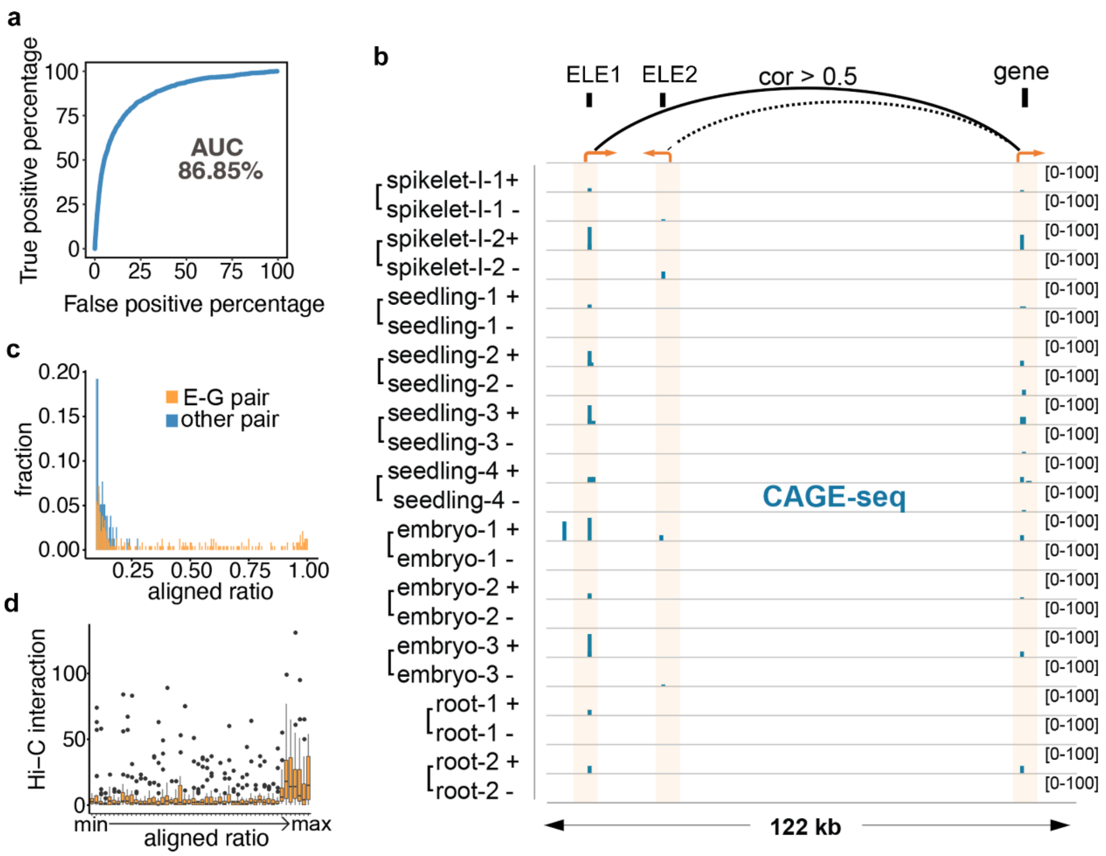

Supplementary Figure 13. Identification of active ELE-TSSs and the linkage to target gene-TSSs.

a. ROC curve distinguishing ELE and gene TSSs according to the k-mer and SVM.
b. Genomic tracks illustrating the ELE targets on the basis of proximity or the correlation between the CAGE signals of the ELE-TSS and gene-TSS.
c. Distribution of the fraction of TSS sequences alignable between E–G pairs and randomly selected E–G pairs.
d. Boxplot presenting the Hi-C contact frequency of the TSS pairs with different levels of sequence similarity. Horizontal lines in boxplots show median, hinges show IQR, whiskers show 1.5 × IQR, points beyond 1.5 × IQR past hinge are shown.

Source data are provided as a Source Data file.

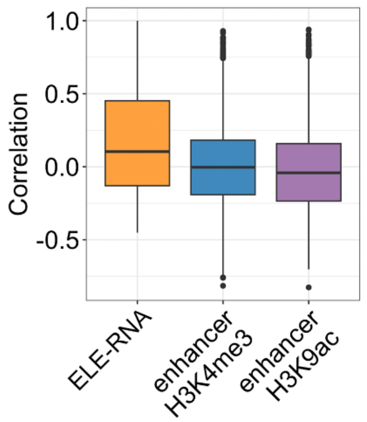

Supplementary Figure 14. Distribution of correlation between ELE activity and the closest gene expression. The activity of ELEs is assessed by the ELE-RNA transcription and the intensities of histone modifications, respectively. The results show that when using ELE-RNA transcription to represent the ELE activity, the correlation is significantly larger (two-tailed Welch Two Sample t-test, p-value< 2.2e-16) than represented by histone modification. Specifically, in the 4339 gene-ELE pairs analyzed, 36% showed an R^2 value greater than 0.5 and a p-value less than 0.05 for the correlation between ELE activity and the closest gene expression. Source data are provided as a Source Data file.

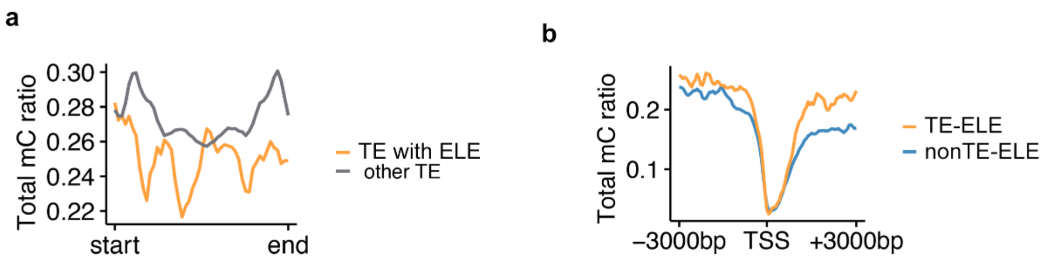

Supplementary Figure 15. DNA methylation profiles surrounding TEs and ELEs derived from TE or nonTE.

a. Distribution of DNA methylation level surrounding full-length TEs with or without ELE-RNA.
b. Distribution of DNA methylation level surrounding 3kb up- and down-stream of ELE-TSS initiated by TEs or within non-TE regions (b).

Source data are provided as a Source Data file.

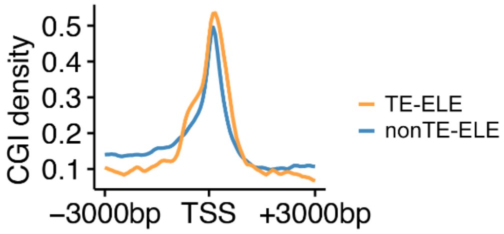

Supplementary Figure 16. The CpG island density in 3 kb up- and down-stream of ELE-TSS derived by TEs or in non-TE regions. Source data are provided as a Source Data file.

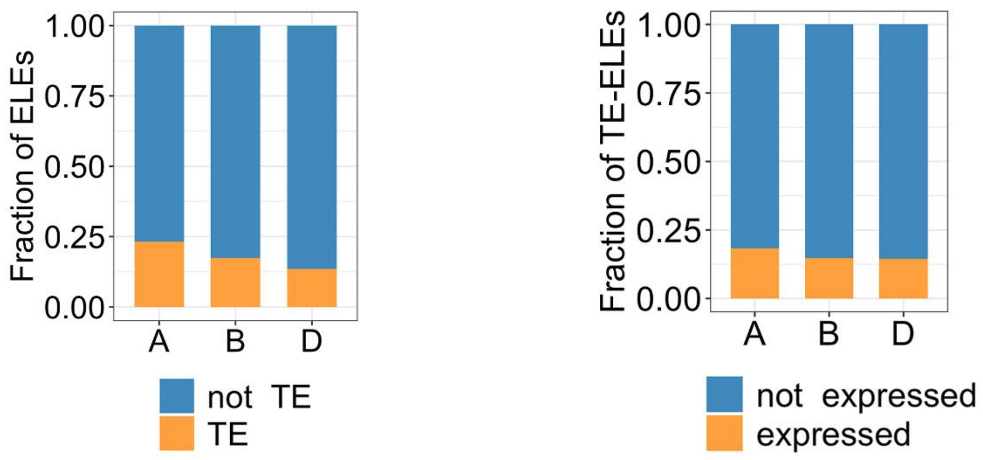

Supplementary Figure 17. The bar plot displays the fraction of TE-derived ELEs (left) and the expressed TE-ELEs (right) among A, B and D subgenomes. Source data are provided as a Source Data file.

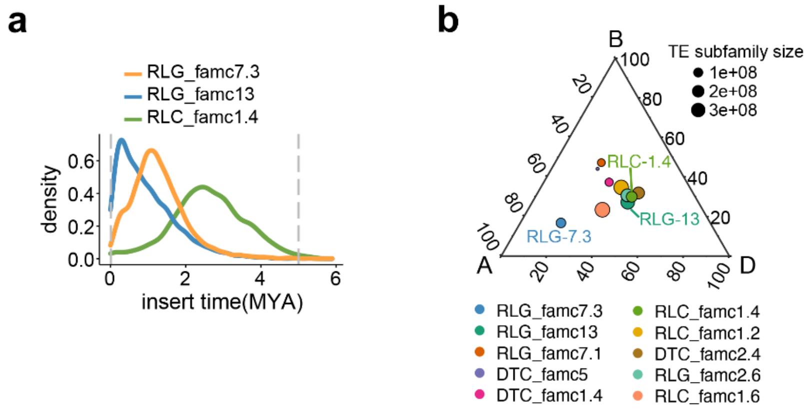

Supplementary Figure 18. The expansion period and abundance of top TE families contributing to TSSs.

a. The age of three LTR-type TEs top contributing to TSS, as reflected by the similarity between two LTR regions. The more similar the LTR regions, the younger the corresponding LTR TEs, since the insertion of new LTR TEs involves the replication of LTR regions[14]. The expansion of three top abundant TEs occurred predominantly in the progenitors of the three subgenomes prior to tetraploidization (~0.5 Mya, vertical dotted lines) after the divergence from A-B-D common ancestor (~5 Mya, vertical dotted lines).

b. Ternary plot presenting the relative abundance of TE subfamilies top contributing to TSS. Each circle represents a TE subfamily. The relative position to the three vertices represents the relative abundance of a given TE subfamily among the three subgenomes. RLG_famc7.3 is predominantly present in subgenome A.

Source data are provided as a Source Data file.

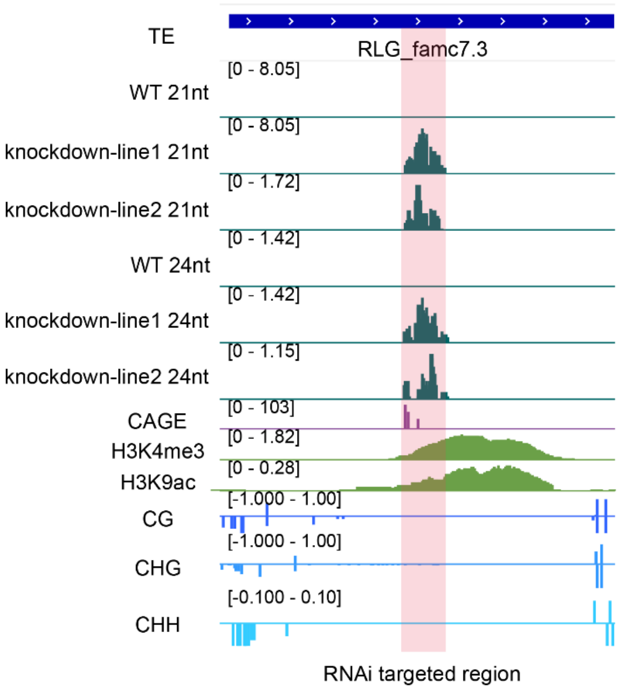

Supplementary Figure 19. Genomic tracks illustrate the 21 and 24nt smRNA profiles generated from wild-type and two independent RNAi knockdown T1 generation materials, along with CAGE-seq and epigenetic signatures in the RLG_famc7.3-ELE region. The targeted region is marked in red shade.

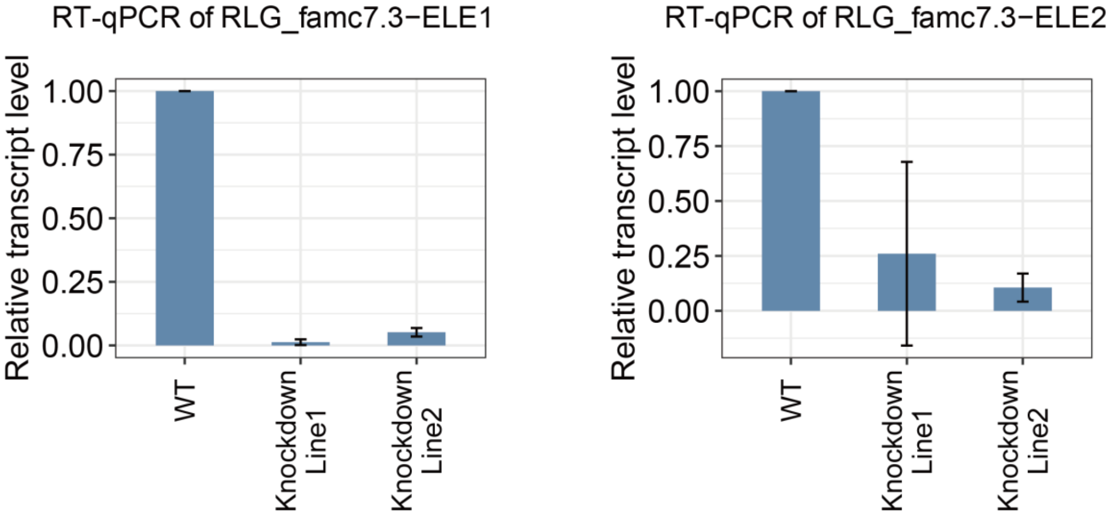

Supplementary Figure 20. Relative transcript level of RLG_famc7.3-ELE-RNA in WT and two RNAi T1 lines quantified by RT-qPCR. All results were normalized to actin (TraesCS1A01G274400). The average values were obtained from three experiments. The length of the error bars represents the standard deviation. Source data are provided as a Source Data file.

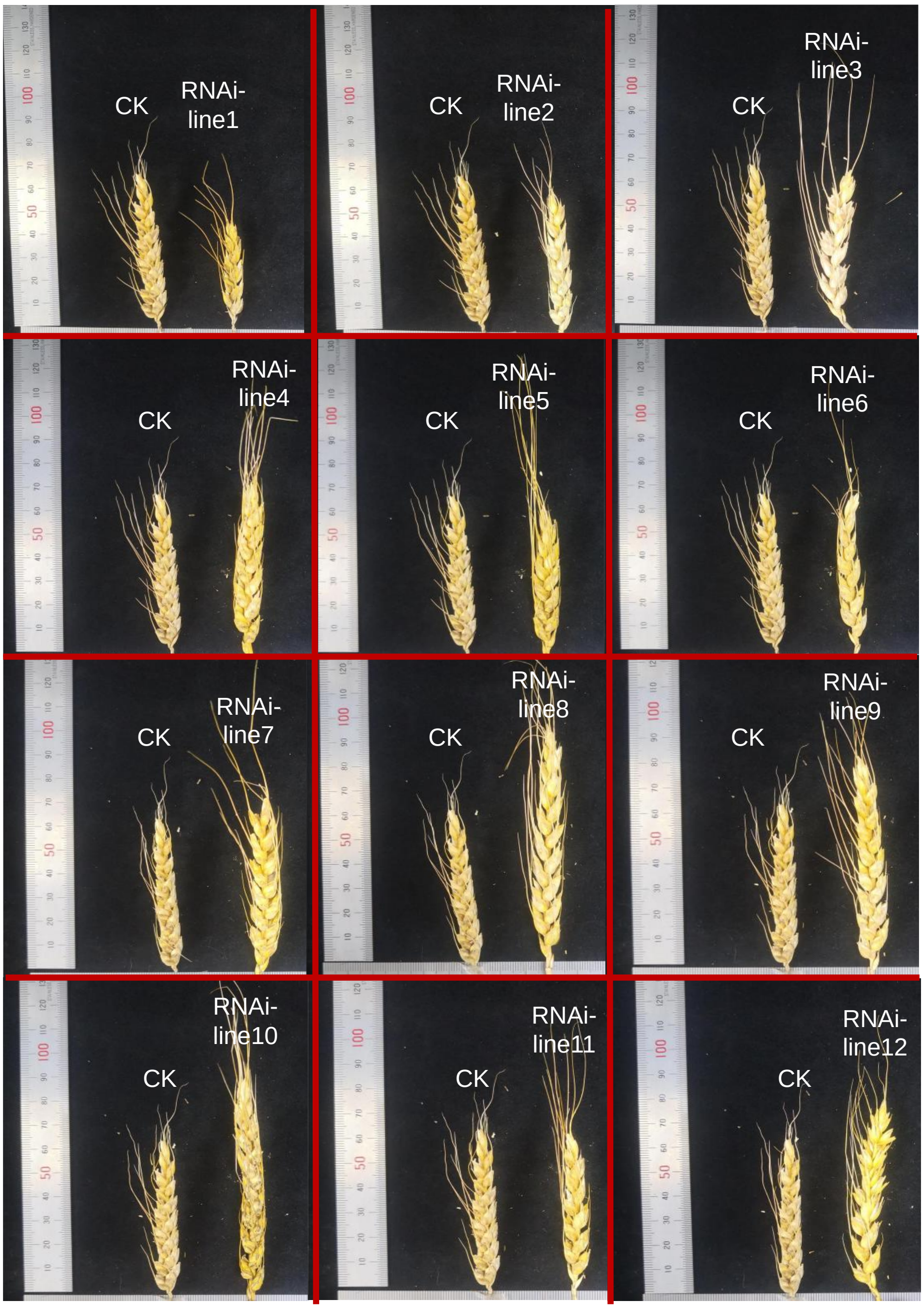

Supplementary Figure 21. Mature spikes of T0 knockdown lines and CKs.

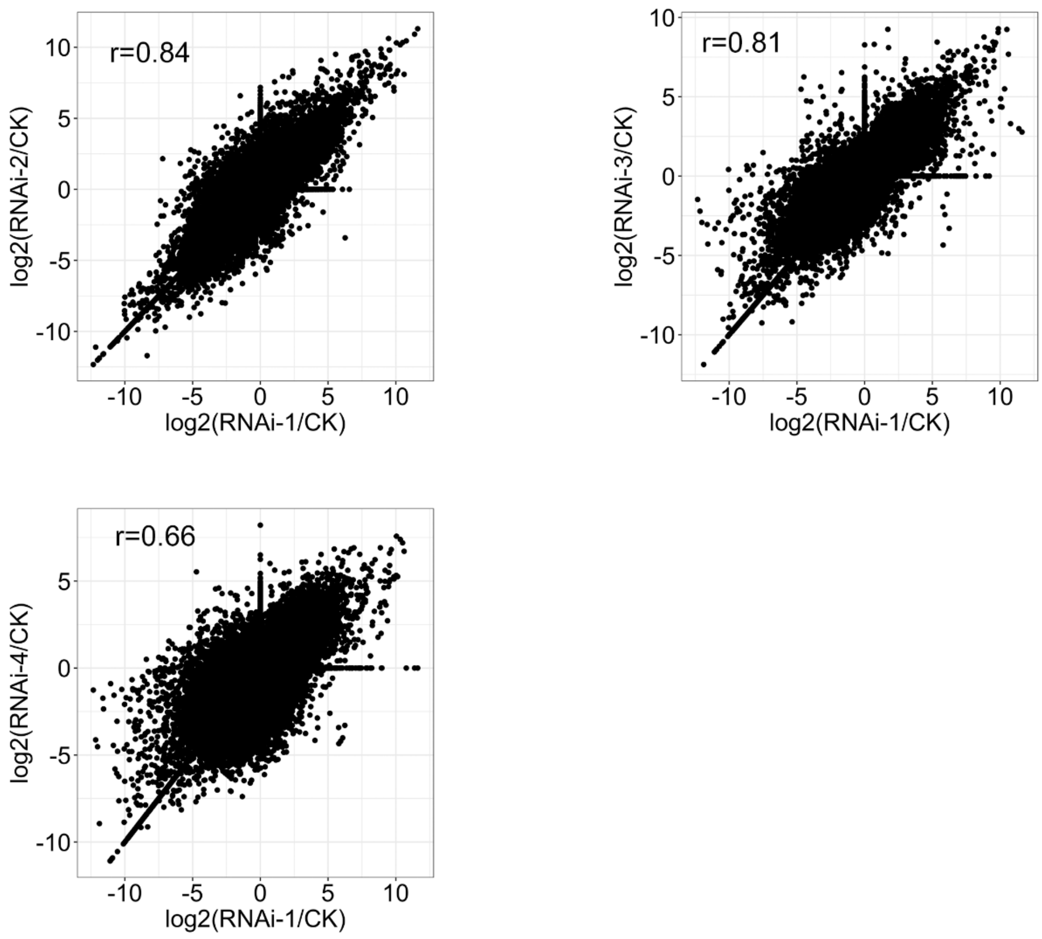

Supplementary Figure 22. The $\log_2$(fold-change) of gene expression between knockdown lines and CK.

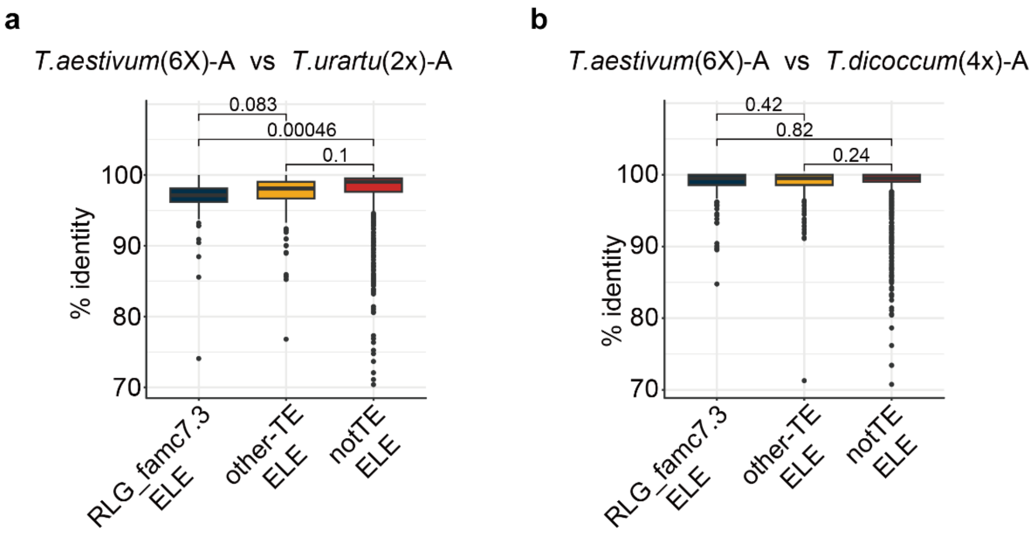

Supplementary Figure 23. Distribution of sequence identity surrounding ELE-TSS in wheat of different ploidy levels.

a. Distribution of sequence identity surrounding ELE-TSS in (sub)genome A between diploid progenitor and hexaploid wheat.
b. The distribution of sequence identity surrounding ELE-TSS in subgenome A between tetraploid and hexaploid common wheat.

Two-tailed Welch Two Sample t-test was used to obtain p-values to assess statistical significance in a-b. Horizontal lines in boxplots show median, hinges show IQR, whiskers show 1.5 × IQR, points beyond 1.5 × IQR past hinge are shown. Source data are provided as a Source Data file.

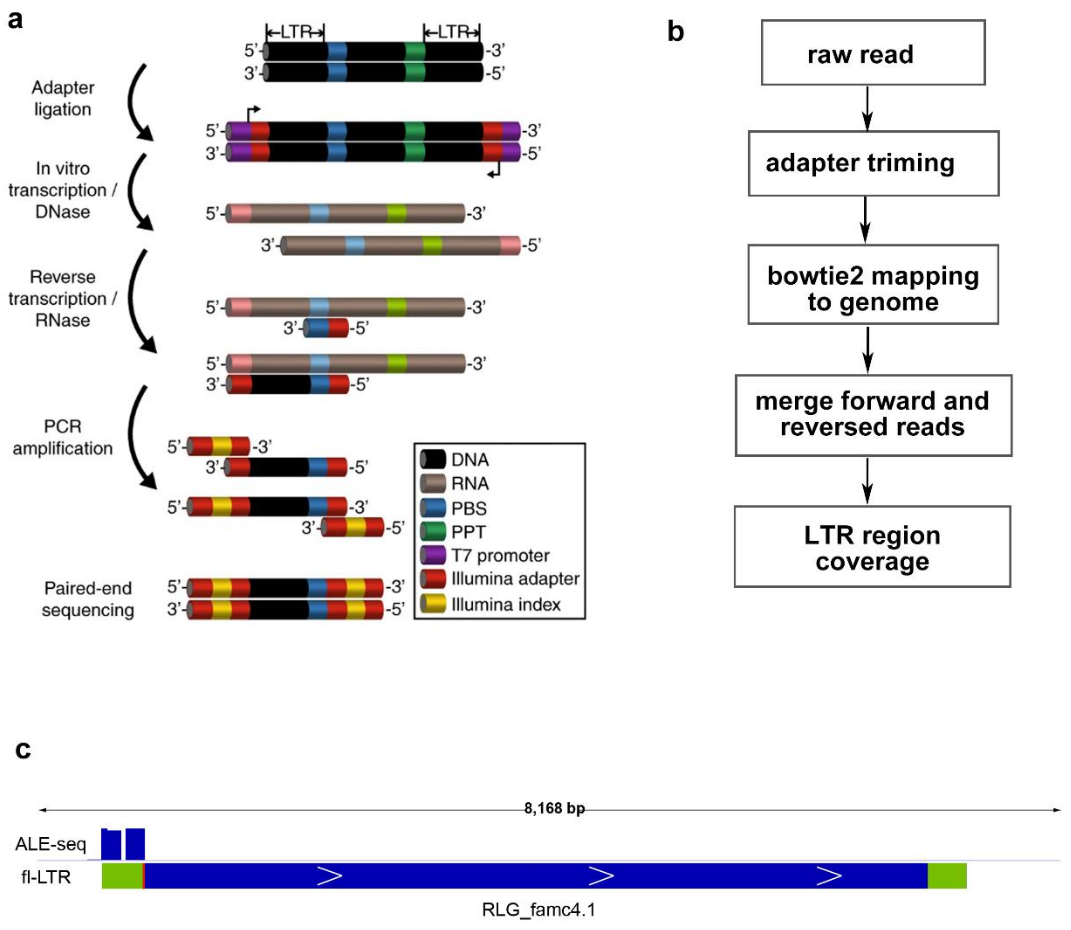

Supplementary Figure 24. The workflow of ALE-seq for detection of eclDNA in bread wheat.

a. The workflow of library construction of ALE-seq, modified from[15].
b. Analysis pipeline of ALE-seq data.
c. The genomic tracks illustrating the coverage of read mapped to a LTR-TE. The green bars represent the LTR region of the TE and red bars represent primer binding site (PBS).

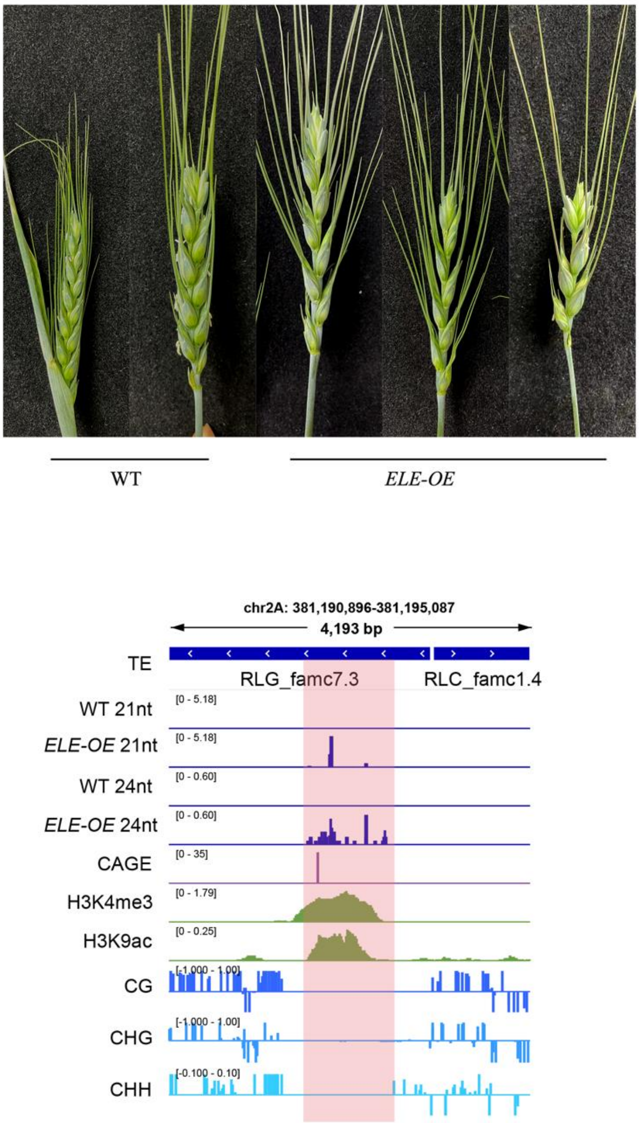

Supplementary Figure 25. Phenotype and epigenetic profiles of ELE-Overexpressed lines. Up: Spikes of WT and T0 ELE-Overexpressed lines. Down: Genomic tracks illustrate the 21 and 24nt smRNA profiles generated from wild-type and one ELE-Overexpressed T0 generation materials, along with CAGE-seq and epi-genetic signatures in the RLG_famc7.3-ELE region. The overexpressed region is marked in red shade.

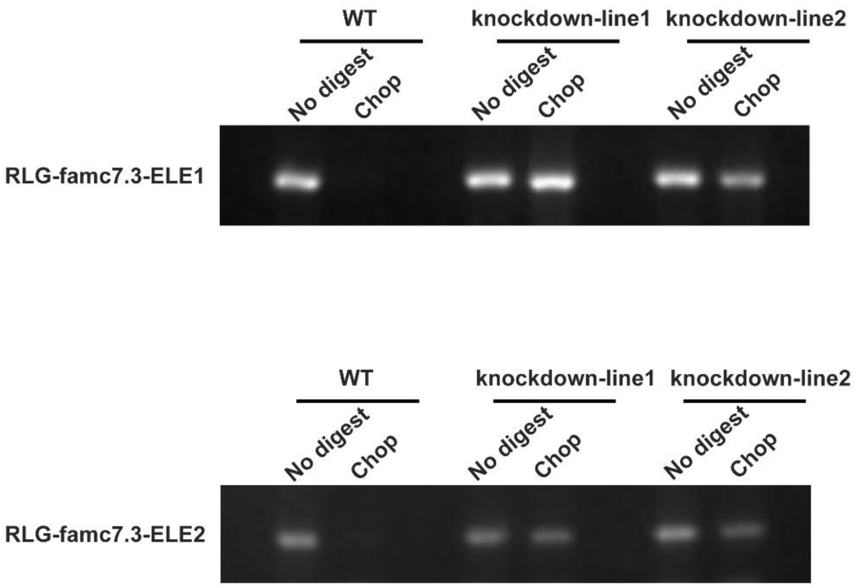

Supplementary Figure 26. Chop-PCR reflects the methylation level of RNAi target loci in two RLG_famc7.3-ELE regions. The T1 generation of two RNAi knockdown lines were used. Chop-PCR is a targeted DNA methylation detection technique that uses partial digestion by methylation-sensitive restriction enzymes (MSREs) followed by PCR amplification. The presence of cytosine methylation at the cleavage sites of the MSREs protects the DNA against digestion and therefore can be amplified using PCR.

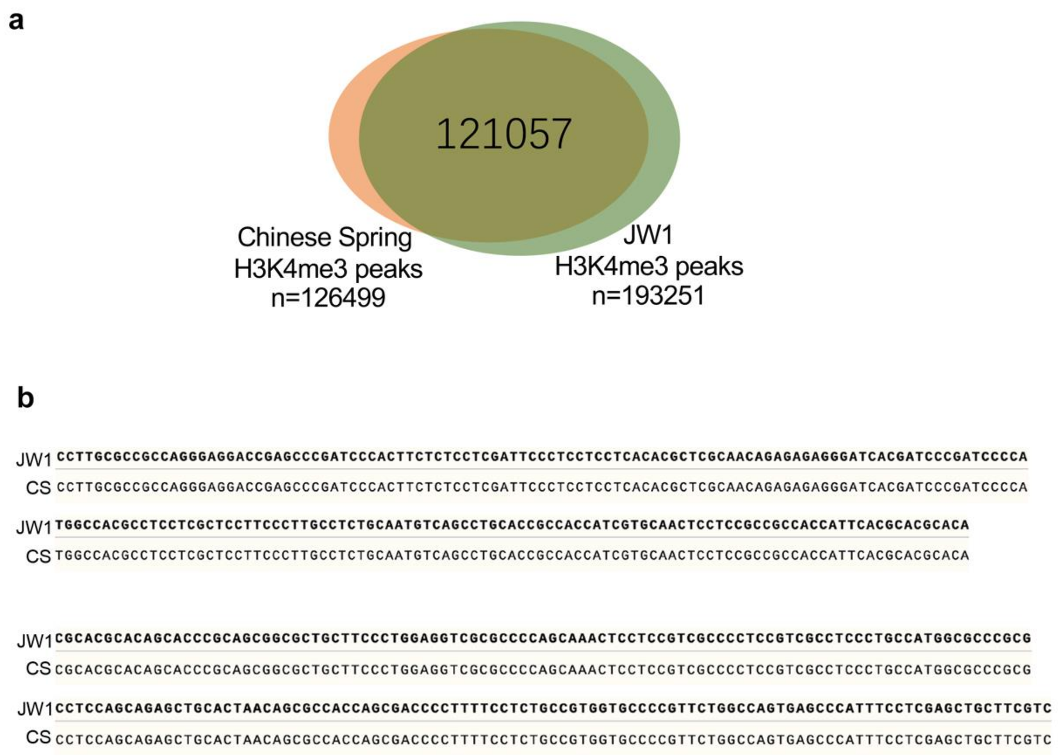

Supplementary Figure 27. The consistency between ‘Chinese Spring’ and ‘JW1’

a. Venn diagram displays the overlap of H3K4me3 peaks between ‘Chinese Spring’ and ‘JW1’.
b. Sequence alignment of RNAi target sequence between ‘Chinese Spring’ and ‘JW1’.

Supplementary Table 1. Primer sequence for 5'RACE .

Gene ID	primer ID	Primer sequence (5'- 3')
/	adaptor-poly（G）	GACGTTCCCGGGTCCGGTACCGGGGGGGGGGGGG
TraesCS2A02G24600	gene-specific-RT-primer	ATCTGCATCGCCTCCTTCTC
	adaptor-nest-PCR primer	TCCGCTAGCCTCGAGGAGCTCGGTCTTACCACAAGCTTTTCCT
TraesCS3A02G041200	gene-specific-RT-primer	CAGCCAGGCATGTCGAGGAAG
	adaptor-nest-PCR primer	TCCGCTAGCCTCGAGGAGCTCCCTCTTGCCGGAGAACG
TraesCS3A02G488500	gene-specific-RT-primer	GTCACCAAATCCTTGGCATGGC
	adaptor-nest-PCR primer	TCCGCTAGCCTCGAGGAGCTCGGATTGAGGTCCAAGACAACGAT

Supplementary Table 2. Reverse transcription primers for ALE-seq.

Primer ID	Primer sequence (5'- 3')
cs_Met_iCAT	AGACGTGTGCTCTTCCGATCTGCTCTGATACCA
cs_ThrAGT	AGACGTGTGCTCTTCCGATCTATGAAAGCACCA
cs_LysCTT	AGACGTGTGCTCTTCCGATCTCGGTGGGCGCCA
cs_PheGAA	AGACGTGTGCTCTTCCGATCTTCAATGAAAGC
cs_ArgCCT	AGACGTGTGCTCTTCCGATCTCCTGGCGCGCCA
cs_ThrCGT	AGACGTGTGCTCTTCCGATCTCCGGGGGCTCC

Supplementary Table 3. Primers for knockdown transgenic lines validation.

Primer ID	Primer sequence (5'- 3')
region1(TSS downstream1-200bp)_F	ATAGTTACGAGTTTAAGATGGATG
region1(TSS downstream1-200bp)_R	GTATATACGTACGGTACCTGTG
region2(TSS downstream200-400bp)_F	ATAGTTACGAGTTTAAGATGGATG
region2(TSS downstream200-400bp)_R	GTATATACGTACGGTACCGACGAAGC

Supplementary Table 4. Phenotyping of knockdown transgenic T1 generation and WT.

ID (line_id-plant_id-spike_id)	Spikelet count	Floret count	Per floret in spikelet	Grain count	Setting rate	Grain weight (g)	Thou-sand kernel weight (g)	Rachis length(cm)	Node length (cm)
38-2-1	20	58	2.900	46	0.793	0.917	19.935	9.662	0.483
38-2-2	16	34	2.125	18	0.529	0.3357	18.650	7.381	0.461
38-1-1	19	73	3.842	8	0.110	0.2074	25.925	8.846	0.466
38-2-3	18	44	2.444	14	0.318	0.3133	22.379	8.388	0.466
38-1-2	8	20	2.500	0	0.000	/	/	3.641	0.455
26-1-2	18	47	2.611	46	0.979	1.2032	26.157	8.706	0.484
26-1-1	16	39	2.438	26	0.667	0.7761	29.850	7.658	0.479
26-1-3	17	49	2.882	41	0.837	0.9778	24.445	8.399	0.494
05-1-1	17	40	2.353	23	0.575	0.512	22.261	7.612	0.448
05-1-2	18	49	2.722	34	0.694	0.727	21.382	8.112	0.451
05-1-3	14	41	2.929	27	0.659	0.5789	21.441	6.627	0.473
57-2-1	15	42	2.800	24	0.571	0.4345	18.104	7.208	0.481
57-2-2	17	49	2.882	36	0.735	0.8691	24.142	7.572	0.445
44-2-1	14	43	3.071	30	0.698	1.0159	33.863	7.251	0.518
44-2-2	15	44	2.933	14	0.318	0.4183	29.879	6.772	0.451
44-2-3	17	45	2.647	27	0.600	0.725	27.885	8.038	0.473
57-1-1	16	52	3.250	33	0.635	1.0203	30.918	6.648	0.416
57-1-2	19	47	2.474	30	0.638	0.569	18.967	5.979	0.315
57-1-3	19	41	2.158	21	0.512	0.388	18.476	6.805	0.358
16-1-1	16	44	2.750	30	0.682	0.7535	25.117	6.672	0.417
16-1-2	13	37	2.846	20	0.541	0.4806	24.030	6.311	0.485
16-1-3	14	32	2.286	17	0.531	0.3935	23.147	6.037	0.431
CK-3-1	15	38	2.533	31	0.816	1.2712	41.006	6.293	0.420
CK-3-2	13	34	2.615	22	0.647	0.8351	37.959	5.951	0.458
CK-3-3	10	25	2.500	0	0.000	/	/	4.349	0.435
CK-2-1	16	41	2.563	29	0.707	0.8853	30.528	5.74	0.359
CK-2-2	14	37	2.643	23	0.622	0.7172	31.183	5.897	0.421

Note: A-B-C: A-CK is WT, and the others are transgenic lines; B - different T1 lines from the same T0 line; C - different spikes from the same plant. Thousand-kernel weight is assessed by weighing the kernels per spike.

Supplementary Table 5. RT-qPCR primers for RLG_famc7.3-ELEs.

**Primer ID**	**Primer sequence (5'- 3')**
ELE-1-F	TGCTTCCCTGGAGGTCGCGC
ELE-1-R	TCACTGGCCAGAACGGGGCA
ELE-2-F	AGCCTCACCACTGGAAGCC
ELE-2-R	GAAGCTGAAGACGTCGGCG
TaActin(TraesCS1A02G274400)-F	ACTTTCCAGCAGATGTGGATCTC
TaActin(TraesCS1A02G274400)-R	TGCCAAACGGAAAAGATGAACCGATAC

Supplementary references

1 Morton, T. *et al.* Paired-end analysis of transcription start sites in Arabidopsis reveals plant-specific promoter signatures. *Plant Cell* **26**, 2746-2760 (2014).

2 Le, N. T. *et al.* Epigenetic regulation of spurious transcription initiation in Arabidopsis. *Nat Commun* **11**, 3224, (2020).

3 Mejía-Guerra, M. K. *et al.* Core promoter plasticity between maize tissues and genotypes contrasts with predominance of sharp transcription initiation sites. *Plant Cell* **27**, 3309-3320 (2015).

4 Wang, K. *et al.* Multi-strategic RNA-seq analysis reveals a high-resolution transcriptional landscape in cotton. *Nat Commun* **10**, 4714 (2019).

5 Demircioglu, D. *et al.* A pan-cancer transcriptome analysis reveals pervasive regulation through alternative promoters. *Cell* **178**, 1465-1477 (2019).

6 Ushijima, T. *et al.* Light controls protein localization through phytochrome-mediated alternative promoter selection. *Cell* **171**, 1316-1325 (2017).

7 Thieffry, A. *et al.* PAMP-triggered genetic reprogramming involves widespread alternative transcription initiation and an immediate transcription factor wave. *Plant Cell* **34**, 2615-2637 (2022).

8 Kurihara, Y. *et al.* Transcripts from downstream alternative transcription start sites evade uORF-mediated inhibition of gene expression in Arabidopsis. *Proc Natl Acad Sci U S A* **115**, 7831-7836 (2018).

9 Wang, M. *et al.* An atlas of wheat epigenetic regulatory elements reveals subgenome divergence in the regulation of development and stress responses. *Plant Cell* **33**, 865-881 (2021).

10 Li, Z. *et al.* The bread wheat epigenomic map reveals distinct chromatin architectural and evolutionary features of functional genetic elements. *Genome Biol* **20**, 139 (2019).

11 Kim, T.-K. *et al.* Widespread transcription at neuronal activity-regulated enhancers. *Nature* **465**, 182-187 (2010).

12 Andersson, R. *et al.* An atlas of active enhancers across human cell types and tissues. *Nature* **507**, 455-461 (2014).

13 Ghandi, M. *et al.* gkmSVM: an R package for gapped-kmer SVM. *Bioinformatics* **32**, 2205-2207 (2016).

14 Wicker, T. *et al.* Impact of transposable elements on genome structure and evolution in bread wheat. *Genome Biol* **19**, 103 (2018).

15 Cho, J. *et al.* Sensitive detection of pre-integration intermediates of long terminal repeat retrotransposons in crop plants. *Nature Plants* **5**, 26-33, (2019).
