# Supplementary material for: Transposable element-initiated enhancer-like elements generate the subgenome-biased spike specificity of polyploid wheat
Source: Nat Commun. 2023 Nov 17;14:7465. doi: 10.1038/s41467-023-42771-9 (PMC10656477; doi:10.1038/s41467-023-42771-9)
Supplement: Supplementary file 3 — Description of Additional Supplementary Files [file 41467_2023_42771_MOESM3_ESM.pdf]

Description of Additional Supplementary Files

Supplementary Data 1

Description: Statistics of sequencing data quality

Supplementary Data 2

Description: TSS clusters identified by CAGE-seq data

Supplementary Data 3

Description: Enhancer TSS cluster and gene pairs which are linked by CAGE-seq signal correlation

Supplementary Data 4

Description: ELE-RNAs specifically expressed in one tissue

Supplementary Data 5

Description: The number of each TE subfamily in each subgenome

Supplementary Data 6

Description: GWAS trait enrichment in SNVs surrounding spikelet-I specific RLG_famc7.3 enhancers

Supplementary Data 7

Description: Statistics of knockdown lines information, include RNA-seq and smRNA-seq data quality, and the primers for Chop-PCR

Supplementary Data 8

Description: Statistics of ALE-seq data
