# Supplementary material for: Transposable element-initiated enhancer-like elements generate the subgenome-biased spike specificity of polyploid wheat
Source: Nat Commun. 2023 Nov 17;14:7465. doi: 10.1038/s41467-023-42771-9 (PMC10656477; doi:10.1038/s41467-023-42771-9)
Supplement: Supplementary file 12 — Reporting Summary [file 41467_2023_42771_MOESM12_ESM.pdf]

Corresponding author(s): Yijing Zhang, Yongbiao Xue, Zhicheng Dong, Jungnan Cho

Last updated by author(s): Oct 10, 2023

Reporting Summary

Nature Portfolio wishes to improve the reproducibility of the work that we publish. This form provides structure for consistency and transparency in reporting. For further information on Nature Portfolio policies, see our Editorial Policies and the Editorial Policy Checklist.

Statistics

For all statistical analyses, confirm that the following items are present in the figure legend, table legend, main text, or Methods section.

n/a	Confirmed	
☐	☒	The exact sample size (*n*) for each experimental group/condition, given as a discrete number and unit of measurement
☐	☒	A statement on whether measurements were taken from distinct samples or whether the same sample was measured repeatedly
☐	☒	The statistical test(s) used AND whether they are one- or two-sided<br>*Only common tests should be described solely by name; describe more complex techniques in the Methods section.*
☒	☐	A description of all covariates tested
☒	☐	A description of any assumptions or corrections, such as tests of normality and adjustment for multiple comparisons
☐	☒	A full description of the statistical parameters including central tendency (e.g. means) or other basic estimates (e.g. regression coefficient) AND variation (e.g. standard deviation) or associated estimates of uncertainty (e.g. confidence intervals)
☐	☒	For null hypothesis testing, the test statistic (e.g. *F*, *t*, *r*) with confidence intervals, effect sizes, degrees of freedom and *P* value noted<br>*Give P values as exact values whenever suitable.*
☒	☐	For Bayesian analysis, information on the choice of priors and Markov chain Monte Carlo settings
☒	☐	For hierarchical and complex designs, identification of the appropriate level for tests and full reporting of outcomes
☒	☐	Estimates of effect sizes (e.g. Cohen's *d*, Pearson's *r*), indicating how they were calculated

Our web collection on statistics for biologists contains articles on many of the points above.

Software and code

Policy information about availability of computer code

Data collection	CAGE-seq libraries were sequenced with Illumina NovaSeq 6000 system.<br>RNA-seq, ChIP-seq and Bisulfite-seq libraries were sequenced with the HiSeq X Ten system<br>smRNA-seq libraries were sequenced with the Illumina NovaSeq SE50
Data analysis	github:https://github.com/yilinlinyi/wheat_ELE_spike;<br>Cutadapt (version 1.18); SortMeRNA (version 2.1b); Bowtie2 (version 2.3.5); R package CAGEr (version 1.28.0); chrHMM (version 1.23); Trim Galore (version 0.6.4); HISAT2 (version2.1.0); Subread package (version1.5.3); Transdecoder (version 5.5.0); hmmsearch (version 3.2.1); bwa (version 0.7.17-r1188); MACS2 (version 2.1.1); Bismark (version0.19.0); LTRharvest (version 1.5.9); dismat (version 6.6.0.0); R package gkmSVM(version 0.80.0); Juicer(version 1.21.01);MCScanX; prank (v.170427); R package DEseq2 (version 1.38.3); tringTie program (version 2.1.4); MEME software toolkit (version 5.1.1); prank ((version 170427);HiC-Pro (version 2.11.1); Homer (version 4.11)

For manuscripts utilizing custom algorithms or software that are central to the research but not yet described in published literature, software must be made available to editors and reviewers. We strongly encourage code deposition in a community repository (e.g. GitHub). See the Nature Portfolio guidelines for submitting code & software for further information.

Data

Policy information about availability of data

All manuscripts must include a data availability statement. This statement should provide the following information, where applicable:

- Accession codes, unique identifiers, or web links for publicly available datasets
- A description of any restrictions on data availability
- For clinical datasets or third party data, please ensure that the statement adheres to our policy

The datasets generated during the current study include CAGE-seq, RNA-seq, ChIP-seq, Bisulfite-seq, smRNA-seq and ALE-seq. The sequencing data are available in the Gene Expression Omnibus (GEO) repository https://www.ncbi.nlm.nih.gov/geo/ under accession number GSE198284 (https://www.ncbi.nlm.nih.gov/geo/query/acc.cgi?acc=GSE198284). Other datasets including Chinese Spring Hi-C data, RNA-seq, ChIP-seq and Bisulfite-seq of Chinese Spring seedlings, nascent RNA sequencing, RNA-seq of Chinese Spring tissues during development and under treatments were published previously.

Research involving human participants, their data, or biological material

Policy information about studies with human participants or human data. See also policy information about sex, gender (identity/presentation), and sexual orientation and race, ethnicity and racism.

Reporting on sex and gender	This article does not involve human research.
Reporting on race, ethnicity, or other socially relevant groupings	This article does not involve human research.
Population characteristics	This article does not involve human research.
Recruitment	This article does not involve human research.
Ethics oversight	This article does not involve human research.

Note that full information on the approval of the study protocol must also be provided in the manuscript.

Field-specific reporting

Please select the one below that is the best fit for your research. If you are not sure, read the appropriate sections before making your selection.

- [x] Life sciences
- [ ] Behavioural & social sciences
- [ ] Ecological, evolutionary & environmental sciences

For a reference copy of the document with all sections, see nature.com/documents/nr-reporting-summary-flat.pdf

Life sciences study design

All studies must disclose on these points even when the disclosure is negative.

Sample size	No statistics method is used to predetermine sample size for CAGE-seq or other related high throughput sequencing. We aimed to investigate the expression and epigenetic dynamics during wheat development, and we selected typical developmental stages of wheat, including vegetative growth, reproductive growth, above-ground parts, and below-ground parts, which are sufficiently representative.
Data exclusions	No data was excluded from the analysis.
Replication	Replicated assays were performed and reproduced the main results in this study (Supplementary data1, Supplementary data7 and Fig. S22).
Randomization	Because we did not have group allocation and perform different treatments in this study, the randomization was not used.
Blinding	Because we did not have group allocation in this study, the blinding was not used.

Reporting for specific materials, systems and methods

We require information from authors about some types of materials, experimental systems and methods used in many studies. Here, indicate whether each material, system or method listed is relevant to your study. If you are not sure if a list item applies to your research, read the appropriate section before selecting a response.

Materials & experimental systems

n/a	Involved in the study
☐	☒ Antibodies
☒	☐ Eukaryotic cell lines
☒	☐ Palaeontology and archaeology
☒	☐ Animals and other organisms
☒	☐ Clinical data
☒	☐ Dual use research of concern
☒	☐ Plants

Methods

n/a	Involved in the study
☐	☒ ChIP-seq
☒	☐ Flow cytometry
☒	☐ MRI-based neuroimaging

Antibodies

Antibodies used

H3K4me3:Abcam-ab8580,Cambridge, England;
H3K9ac:Millipore-07352,Upstate, USA;
H3K27me3:Millipore-07360,Upstate, USA

Validation

The antibody has been validated by the supplier. The reports could be found on the the supplier website.

Plants

Seed stocks

We used Common wheat (Triticum aestivum variety 'Chinese Spring' and 'JW1') in this study and generated the transgenic lines by our own.

Novel plant genotypes

The sequences downstream 1-200 bp and 200-400bp of the RLG_famc7.3 ELE- TSS located in JW1 and have identical sequences in CS (Fig. S25b) were synthesized and cloned into pEXT06/g to construct RNAi silencing vector. The plasmid was then transformed into wheat cultivar ‘JW1’ via Agrobacterium mediated transformation by Wimi Biotechnology (http://www.wimibio.com/). The primer sequences used in this study are shown in Table S11.

Authentication

Enhancer RNAs are generally detected based on nascent RNA sequencing methods. RNAi occurs at post-transcriptional level. To examine the effect of RNAi, we performed small RNA sequencing and detected a significant accumulation of small RNAs surrounding RLG_famc7.3-initiated eRNAs (Fig. S19).

ChIP-seq

Data deposition

☒ Confirm that both raw and final processed data have been deposited in a public database such as GEO.

☒ Confirm that you have deposited or provided access to graph files (e.g. BED files) for the called peaks.

Data access links
May remain private before publication.

https://www.ncbi.nlm.nih.gov/geo/ under accession number GSE198284 (reviewer token utibgauoxfanhcr)

Files in database submission

100mix_ale_rep1.bw;100mix_ale_rep1.R1.fq.gz;100mix_ale_rep1.R2.fq.gz;33.1_5_ale_rep1.bw;33.1_5_ale_rep1.R1.fq.gz;33.1_5_ale_rep1.R2.fq.gz;33.3_5_ale_rep1.bw;33.3_5_ale_rep1.R1.fq.gz;33.3_5_ale_rep1.R2.fq.gz;33.3_6_ale_rep1.bw;33.3_6_ale_rep1.R1.fq.gz;33.3_6_ale_rep1.R2.fq.gz;AA_G1218_embryo_rep1_R1.fq.gz;AA_G1218_embryo_rep1_R2.fq.gz;AA_G1218_embryo_rep2_R1.fq.gz;AA_G1218_embryo_rep2_R2.fq.gz;AA_G1812_cage_embryo_rep1_unique_best.site_frq.bed;AA_G1812_cage_embryo_rep2_unique_best.site_frq.bed;cs_anther_ale_rep1.bw;cs_anther_ale_rep1.R1.fq.gz;cs_anther_ale_rep1.R2.fq.gz;cs_bisulfite_embryo_001_R1.fq.gz;cs_bisulfite_embryo_001_R2.fq.gz;cs_bisulfite_embryo_002_R1.fq.gz;cs_bisulfite_embryo_002_R2.fq.gz;cs_bisulfite_root_001_R1.fq.gz;cs_bisulfite_root_001_R2.fq.gz;cs_bisulfite_root_002_R1.fq.gz;cs_bisulfite_root_002_R2.fq.gz;cs_bisulfite_spikeletI_001_R1.fq.gz;cs_bisulfite_spikeletI_001_R2.fq.gz;cs_bisulfite_spikeletI_002_R1.fq.gz;cs_bisulfite_spikeletI_002_R2.fq.gz;cs_cage_embryo_rep1_1_R1.fq.gz;cs_cage_embryo_rep1_1_R2.fq.gz;cs_cage_embryo_rep1_2_R1.fq.gz;cs_cage_embryo_rep1_2_R2.fq.gz;cs_cage_embryo_rep1_unique_best.site_frq.bed;cs_cage_embryo_rep2_1_R1.fq.gz;cs_cage_embryo_rep2_1_R2.fq.gz;cs_cage_embryo_rep2_2_R1.fq.gz;cs_cage_embryo_rep2_2_R2.fq.gz;cs_cage_embryo_rep2_unique_best.site_frq.bed;cs_cage_embryo_rep3_1_R1.fq.gz;cs_cage_embryo_rep3_1_R2.fq.gz;cs_cage_embryo_rep3_unique_best.site_frq.bed;cs_cage_root_rep1_1_R1.fq.gz;cs_cage_root_rep1_1_R2.fq.gz;cs_cage_root_rep1_unique_best.site_frq.bed;cs_cage_root_rep2_1_R1.fq.gz;cs_cage_root_rep2_1_R2.fq.gz;cs_cage_root_rep2_unique_best.site_frq.bed;cs_cage_seedling_rep1_1_R1.fq.gz;cs_cage_seedling_rep1_1_R2.fq.gz;cs_cage_seedling_rep1_2_R1.fq.gz;cs_cage_seedling_rep1_2_R2.fq.gz;cs_cage_seedling_rep1_unique_best.site_frq.bed;cs_cage_seedling_rep2_1_R1.fq.gz;cs_cage_seedling_rep2_unique_best.site_frq.bed;cs_cage_seedling_rep3_1_R1.fq.gz;cs_cage_seedling_rep3_1_R2.fq.gz;cs_cage_seedling_rep3_2_R1.fq.gz;cs_cage_seedling_rep3_2_R2.fq.gz;cs_cage_seedling_rep3_unique_best.site_frq.bed;cs_cage_seedling_rep4_1_R1.fq.gz;cs_cage_seedling_rep4_1_R2.fq.gz;cs_cage_seedling_rep4_unique_best.site_frq.bed;cs_cage_spikelet_I_rep1_1_R1.fq.gz;cs_cage_spikelet_I_rep1_1_R2.fq.gz;cs_cage_spikelet_I_rep1_2_R1.fq.gz;cs_cage_spikelet_I_rep1_2_R2.fq.gz;cs_cage_spikelet_I_rep1_unique_best.site_frq.bed;cs_cage_spikelet_I_rep2_1_R1.fq.gz;cs_cage_spikelet_I_rep2_1_R2.fq.gz;cs_cage_spikelet_I_rep2_2_R1.fq.gz;cs_cage_spikelet_I_rep2_2_R2.fq.gz;cs_cage_spikelet_I_rep2_unique_best.site_frq.bed;cs_callus_ale_rep1.bw;cs_callus_ale_rep1.R1.fq.gz;cs_callus_ale_rep1.R2.fq.gz;cs_CK1_rnaseq_FPKM.txt;cs_CK1_rnaseq_R1.fq.gz;cs_CK1_rnaseq_R2.fq.gz;cs_CK2_rnaseq_FPKM.txt;cs_CK2_rnaseq_R1.fq.gz;cs_CK2_rnaseq_R2.fq.gz;cs_CK3_rnaseq_FPKM.txt;cs_CK3_rnaseq_R1.fq.gz;cs_CK3_rnaseq_R2.fq.gz;cs_em_ale_rep1.bw;cs_em_ale_rep1.R1.fq.gz;cs_em_al

e_rep1.R2.fq.gz;cs_em_ale_rep2.bw;cs_em_ale_rep2.R1.fq.gz;cs_em_ale_rep2.R2.fq.gz;cs_em_ale_rep3.bw;cs_em_ale_rep3.R1.fq.gz;cs_em_ale_rep3.R2.fq.gz;cs_embryo_CG_ratio.bw;cs_embryo_CHG_ratio.bw;cs_embryo_CHH_ratio.bw;CS_embryo_H3K27me3_rep1.bw;CS_embryo_H3K27me3_rep1_R1.fq.gz;CS_embryo_H3K27me3_rep1_R2.fq.gz;CS_embryo_H3K4me3_rep1.bw;CS_embryo_H3K4me3_rep1_R1.fq.gz;CS_embryo_H3K4me3_rep1_R2.fq.gz;CS_embryo_H3K9ac_rep1.bw;CS_embryo_H3K9ac_rep1_R1.fq.gz;CS_embryo_H3K9ac_rep1_R2.fq.gz;cs_endosperm_ale_rep1.bw;cs_endosperm_ale_rep1.R1.fq.gz;cs_endosperm_ale_rep1.R2.fq.gz;cs_heat_ale_rep1.bw;cs_heat_ale_rep1.R1.fq.gz;cs_heat_ale_rep1.R2.fq.gz;CS_RNA_embryo_rep1.R1.fq.gz;CS_RNA_embryo_rep1.R2.fq.gz;cs_RNA_embryo_rep1_readcount.txt;cs_RNAi1_rnaseq_FPKM.txt;cs_RNAi1_rnaseq_R1.fq.gz;cs_RNAi1_rnaseq_R2.fq.gz;cs_RNAi2_rnaseq_FPKM.txt;cs_RNAi2_rnaseq_R1.fq.gz;cs_RNAi2_rnaseq_R2.fq.gz;cs_RNAi3_rnaseq_FPKM.txt;cs_RNAi3_rnaseq_R1.fq.gz;cs_RNAi3_rnaseq_R2.fq.gz;cs_RNAi4_rnaseq_FPKM.txt;cs_RNAi4_rnaseq_R1.fq.gz;cs_RNAi4_rnaseq_R2.fq.gz;CS_RNA_root_rep1_1.R1.fq.gz;CS_RNA_root_rep1_1.R2.fq.gz;CS_RNA_root_rep1_2.R1.fq.gz;CS_RNA_root_rep1_2.R2.fq.gz;cs_RNA_root_rep1_readcount.txt;cs_root_50um_ale_rep1.bw;cs_root_50um_ale_rep1.R1.fq.gz;cs_root_50um_ale_rep1.R2.fq.gz;cs_root_ale_rep1.bw;cs_root_ale_rep1.R1.fq.gz;cs_root_ale_rep1.R2.fq.gz;cs_root_CG_ratio.bw;cs_root_CHG_ratio.bw;cs_root_CHH_ratio.bw;CS_root_H3K27me3_rep1.bw;CS_root_H3K27me3_rep1_R1.fq.gz;CS_root_H3K27me3_rep1_R2.fq.gz;CS_root_H3K4me3_rep1.bw;CS_root_H3K4me3_rep1_R1.fq.gz;CS_root_H3K4me3_rep1_R2.fq.gz;CS_root_H3K9ac_rep1_1_R1.fq.gz;CS_root_H3K9ac_rep1_1_R2.fq.gz;CS_root_H3K9ac_rep1_2_R1.fq.gz;CS_root_H3K9ac_rep1_2_R2.fq.gz;CS_root_H3K9ac_rep1.bw;cs_seedling_ale_rep2.bw;cs_seedling_ale_rep2.R1.fq.gz;cs_seedling_ale_rep2.R2.fq.gz;cs_spikelet_I_ale_rep1.bw;cs_spikelet_I_ale_rep1.R1.fq.gz;cs_spikelet_I_ale_rep1.R2.fq.gz;cs_spikelet_I_CG_ratio.bw;cs_spikelet_I_CHG_ratio.bw;cs_spikelet_I_CHH_ratio.bw;cs_style_ale_rep1.bw;cs_style_ale_rep1.R1.fq.gz;cs_style_ale_rep1.R2.fq.gz;F2_2_ale_rep1.bw;F2_2_ale_rep1.R1.fq.gz;F2_2_ale_rep1.R2.fq.gz;G1812_seedling_ale_rep1.bw;G1812_seedling_ale_rep1.R1.fq.gz;G1812_seedling_ale_rep1.R2.fq.gz;TAA_ale_rep1.bw;TAA_ale_rep1.R1.fq.gz;TAA_ale_rep1.R2.fq.gz;XX329_ale_rep1.bw;XX329_ale_rep1.R1.fq.gz;XX329_ale_rep1.R2.fq.gz;CK_srna_seq.fq.gz;RNAi1_srna_seq.fq.gz;RNAi2_srna_seq.fq.gz;CK_21nt.rpm.bw;RNAi1_21nt.rpm.bw;RNAi2_21nt.rpm.bw ;
CK_24nt.rpm.bw;RNAi1_24nt.rpm.bw;RNAi2_24nt.rpm.bw;JW1_spikelet_H3K4me3.R1.fastq.gz;JW1_spikelet_H3K4me3.R2.fastq.gz;JW1_spikelet_H3K9ac.R1.fastq.gz;JW1_spikelet_H3K9ac.R2.fastq.gz;JW1_spikelet_H3K4me3.peaks.bed;JW1_spikelet_H3K9ac.peaks.bed

Genome browser session
(e.g. UCSC)

http://119.78.67.240/JBrowse/?data=cs_eRNA_jbrowse

Methodology

Replicates

cs_cage_seedling_rep1;cs_cage_seedling_rep2;cs_cage_seedling_rep3;cs_cage_seedling_rep4;cs_cage_spikelet_I_rep1;cs_cage_spikelet_I_rep2;cs_cage_embryo_rep1;cs_cage_embryo_rep2;cs_cage_embryo_rep3;cs_cage_root_rep1;cs_cage_root_rep2, the correlations of the replicates are in Supplementary table S1.

Sequencing depth

All CAGE-seq, Bisulfite-seq, ChIP-seq and RNA-seq reads were paired-end 150bp. The number of raw read pairs were show below:
cs_cage_embryo_rep1 92265264
cs_cage_embryo_rep2 19384074
cs_cage_embryo_rep3 104005883
cs_cage_seedling_rep1 108808271
cs_cage_seedling_rep2 48712475
cs_cage_seedling_rep3 62332460
cs_cage_seedling_rep4 98353976
cs_cage_spikelet-I_rep1 58944069
cs_cage_spikelet-I_rep2 130329136
cs_cage_root_rep1 88008314
cs_cage_root_rep2 92517760
cs_RNA_root_rep1 57368754
cs_RNA_root_rep2 37533142 (download from SRR947016)
cs_RNA_root_rep3 29313406 (download from SRR946457)
cs_RNA_embryo_rep1 63407747
cs_RNA_embryo_rep2 15875235 (download from SRR12103282)
cs_RNA_embryo_rep3 16018485 (download from SRR12103286)
cs_RNA_seedling_rep1 62846861 (download from SRR9360460)
cs_RNA_seedling_rep2 95368714 (download from SRR10300719)
cs_RNA_spikelet_I_rep1 42137897 (download from SRR10300726)
cs_RNA_spikelet_I_rep2 52465585 (download from SRR10300727)
cs_bisulfite_embryo 605039974
cs_bisulfite_spikelet_I 645520959
cs_bisulfite_root 596113514
Bisulfite-seq of seedling data are downloaded from SRP133674
CS_embryo_H3K4me3_rep1 93189114
CS_embryo_H3K9ac_rep1 101202286
CS_embryo_H3K27me3_rep1 146247100
CS_root_H3K4me3_rep1 217364552
CS_root_H3K9ac_rep1 149819940
CS_root_H3K27me3_rep1 159823740
ChIP-seq of seedling and spikelet_I data are download from GSE139019
cs_anther_ale_rep1 15694262
cs_callus_ale_rep1 16700315
cs_endosperm_ale_rep1 12534929
cs_root_ale_rep1 12928691

cs_seedling_ale_rep2 14418994
cs_spikelet_I_ale_rep1 11488879
cs_style_ale_rep1 11325387
cs_embryo_ale_rep1 15328845
cs_embryo_ale_rep2 18325406
cs_embryo_ale_rep3 16483134
100mix_ale_rep1 14811039
33.1_5_ale_rep1 19313008
33.3_5_ale_rep1 22510203
33.3_6_ale_rep1 19272644
cs_heat_ale_rep1 20904957
cs_root_50um_5az_ale_rep1 13652849
F2_2_ale_rep1 14780333
TAA_ale_rep1 9979455
XX329_ale_rep1 13726583
G1812_seedling_ale_rep1 18053223
cs_CK1_rnaseq 7178217
cs_CK2_rnaseq 13830859
cs_CK3_rnaseq 13816932
cs_knockdown1_rnaseq 11670667
cs_knockdown2_rnaseq 12958959
cs_knockdown3_rnaseq 14632968
cs_knockdown4_rnaseq 16510101
JW1_spikelet_H3K4me3 48679466
JW1_spikelet_H3K9ac 46456598

Antibodies

H3K4me3:Abcam-ab8580,Cambridge, England;
H3K9ac:Millipore-07352,Upstate, USA;
H3K27me3:Millipore-07360,Upstate, USA

Peak calling parameters

MACS2 was used with parameters: "callpeak -f BAMPE -g 14271578887 --nomodel --nolambda" , FDR < 0.05 and P value < 1e-10.

Data quality

The Peaks numbers of the ChIP-seq generated in this article are listed below:
CS_embryo_H3K4me3_rep1 132696
CS_embryo_H3K9ac_rep1 100547
CS_embryo_H3K27me3_rep1 19987
CS_root_H3K4me3_rep1 140974
CS_root_H3K9ac_rep1 73272
CS_root_H3K27me3_rep1 123438
JW1_spikelet_H3K4me3 193251
JW1_spikelet_H3K9ac 343721

Software

Trim Galore (version 0.6.4); bwa (version 0.7.17-r1188); MACS2 (version 2.1.1); MCScanX; prank (v.170427); MEME software toolkit (version 5.1.1)
